# Clinical Characteristics and Factors Associated With Severe Outcomes of 1891 Pediatric Patients Admitted to the Referral Cholera Treatment Centers in Lusaka, Zambia, December 2023–March 2024

**DOI:** 10.1093/ofid/ofaf215

**Published:** 2025-04-09

**Authors:** Nawa Kalima, Tadatsugu Imamura, Ilunga Chambah, Nyuma Mbewe, Annel Sinkala, Chalilwe Chungu, Khozya Zyambo, Paul Msanzya Zulu, Kelvin Mwangilwa, Muzala Kapin’a, Anchingika Mugala, Kabaso Mwewa, Paul Mashanga, Natasha Ngwenya, Bob Chirwa, Shingo Mitsushima, Yuuki Tsuchihashi, Taro Kamigaki, Aggrey Mweemba, Nathan Kapata, Roma Chilengi, Lloyd Mulenga

**Affiliations:** National Heart Hospital, Lusaka, Zambia; Zambia Paediatric Association, Lusaka, Zambia; Japan International Cooperation Agency, Tokyo, Japan; Center for Postgraduate Education and Training, National Center for Child Health and Development, Tokyo, Japan; Department of Pediatrics, University Teaching Hospital, Lusaka, Zambia; Zambia National Public Health Institute, Lusaka, Zambia; Adult Infectious Diseases Center, University Teaching Hospital, Lusaka, Zambia; Zambia Paediatric Association, Lusaka, Zambia; Catholic Relief Services Zambia, Programming and Health, Lusaka, Zambia; Zambia Paediatric Association, Lusaka, Zambia; Adult Infectious Diseases Center, University Teaching Hospital, Lusaka, Zambia; Zambia National Public Health Institute, Lusaka, Zambia; Zambia National Public Health Institute, Lusaka, Zambia; Zambia National Public Health Institute, Lusaka, Zambia; Levy Mwanawasa University Teaching Hospital, Lusaka, Zambia; Adult Infectious Diseases Center, University Teaching Hospital, Lusaka, Zambia; Levy Mwanawasa University Teaching Hospital, Lusaka, Zambia; Levy Mwanawasa University Teaching Hospital, Lusaka, Zambia; Adult Infectious Diseases Center, University Teaching Hospital, Lusaka, Zambia; Center for Field Epidemic Intelligence, Research and Professional Development, National Institute of Infectious Diseases, Tokyo, Japan; Center for Field Epidemic Intelligence, Research and Professional Development, National Institute of Infectious Diseases, Tokyo, Japan; Center for Surveillance, Immunization, and Epidemiologic Research, National Institute of Infectious Diseases, Tokyo, Japan; Catholic Relief Services Zambia, Programming and Health, Lusaka, Zambia; Zambia National Public Health Institute, Lusaka, Zambia; Zambia National Public Health Institute, Lusaka, Zambia; Adult Infectious Diseases Center, University Teaching Hospital, Lusaka, Zambia

**Keywords:** cholera treatment centers, clinical outcomes, HIV, pediatric patients, severe acute malnutrition

## Abstract

**Background:**

Zambia declared a cholera outbreak on 18 October 2023 and, as of 31 June 2024, had recorded 23 381 cases and 740 deaths. Of the patients seen at the 2 main cholera treatment centers in the capital Lusaka, a third of them were children aged 0 to 15 years. Despite the significant pediatric cholera burden, risk factors for mortality and prolonged hospitalization remain unknown.

**Methods:**

A retrospective data review was conducted by examining the clinical characteristics of patients aged 0 to 15 years hospitalized at the 2 cholera treatment centers between 15 October 2023 and 31 March 2024. Descriptive analysis was conducted for patient characteristics, and penalized logistic regression (PLR) was used to analyze risk factors for the outcomes.

**Results:**

A total of 1891 patients were identified, among which 1.4% (18/1253) had fatal outcomes and 47.9% (399/833) had hospitalization >2 days. By the PLR, the following factors were independently correlated with hospitalization >2 days: HIV infection (odds ratio [OR], 6.89; 95% CI, 1.32–71.9), severe acute malnutrition (SAM; OR, 10.8; 95% CI, 2.91–61.1), and dehydration treatment plans B (OR, 3.93; 95% CI, 1.80–9.27) and C (OR, 7.54; 95% CI, 2.71–22.9). For the fatal outcome, none of them independently showed any significant correlations by the PLR, although younger age and SAM were positively associated by bivariate analysis.

**Conclusions:**

Comorbidities such as SAM and HIV, being on plan B or C, and deteriorating and requiring more intense treatment are associated with longer hospitalization. Risk factors for mortality need to be further investigated.

## Teaser

During the 2023–2024 cholera outbreak in Lusaka, Zambia, characteristics of pediatric patients—including comorbidities (HIV infection, severe acute malnutrition) and being on dehydration treatment plans B and C—were associated with severe clinical outcomes, such as longer hospitalization and mortality.

*Vibrio cholerae* remains a pathogen of public health significance in Africa, with 367 134 cholera cases and 6614 deaths recorded between 2022 and 30 April 2024 [1]. Infection results in symptoms in 20% of cases, of which acute watery diarrhea often leads to varying levels of dehydration, with at least a fifth of such cases having severe dehydration [2]. The acute nature of dehydration can result in a case fatality rate (CFR) of up to 50%, but the response to treatment is often rapid, with CFR reduced to as low as 1% in well-managed outbreaks [2]. Zambia declared an outbreak on 18 October 2023 and, as of 31 June 2024, had recorded 23 381 cases and 740 deaths, representing a combined CFR of 3.17% [3].

In the Zambian outbreak, the first cases and the majority of the cases were from Lusaka district, with a population >2 204 059 as of the 2022 census [4]. Of the patients seen at the 2 main cholera treatment centers (CTCs)—National Heroes Stadium CTC (Heroes CTC) and Levy Mwanawasa University Teaching Hospital CTC (Levy CTC)—a third were children aged 0 to 15 years. Studies in cholera-endemic countries show more cholera positivity among children aged <5 years than any other age group [5]. However, the CFR among the pediatric patients varies among authors, with higher CFRs in some publications and lower CFRs in others, as shown by a recent scoping review [6]. In managing cholera in children, agency, quick response, judicious administration of fluids, and close monitoring are often the difference between life and death. Other possible risk factors identified in literature that may contribute to pediatric cholera morbidity and mortality include comorbidities and nutritional status [6]. Yet, there still exists a limited number of studies on children focusing on these risk factors in our setting, and we found none examining treatment progression and duration of hospitalization.

In this study, we aim to describe the clinical characteristics of pediatric patients hospitalized at the 2 flagship cholera treatment centers in Lusaka and how they affected outcomes.

## METHODS

### Study Design

We retrospectively reviewed the clinical characteristics of pediatric patients who were hospitalized between 15 October 2023 and 31 March 2024 at the 2 main referral cholera treatment centers in Lusaka, Zambia: Heroes CTC and Levy CTC. Levy CTC was opened first on 18 December 2023, followed by Heroes CTC on 4 January 2024 after the overwhelming number of new cholera cases. Both CTCs were part of a centralized approach, with Levy CTC initially managing all patients until Heroes CTC was opened; after which, Levy was dedicated to treating mostly patients with cholera and comorbidities, while Heroes saw only patients without comorbidities and referred comorbid cases to Levy within 24 hours. The Ministry of Health defined comorbidities for referral to Levy CTC as disease conditions that had the potential to influence the choice of care or contribute to morbidity and mortality, thereby requiring specialized care beyond the scope of a standard cholera treatment center. Together, these CTCs catered to patients referred from Lusaka district health facilities and the nearby Chilanga district. At a capacity of 1500 bed spaces, Heroes was the largest CTC in the country, while Levy had 200 bed spaces. At admission, patients with cholera were first assessed for disease severity and then diagnosed as follows: *no dehydration*—awake and alert, normal pulse and thirst, eyes not sunken, skin pinch normal; *some dehydration*—at least 2 of the following: irritable or restless, sunken eyes, rapid pulse, thirsty, skin pinch goes back slowly; or *severe dehydration—*1 or more danger signs: lethargic or unconscious, absent or weak pulse, respiratory distress; or at least 2 of the following: not able to drink or drinks poorly, skin pinch goes back very slowly, sunken eyes [7]. Patients with no, some, and severe dehydration were assigned with treatment plans A (oral rehydration solution over 4 hours), B (oral rehydration solution over 4 hours, 75 mL/kg), and C (intravenous Ringer lactate), respectively. If the patients had a comorbidity of severe acute malnutrition (SAM)—defined as mid upper arm circumference <11.5 cm (only for age 6–59 months), weight-for-height *z* score <−3, or bilateral pitting edema—modified treatment plans A to C (eg, low-osmolarity oral rehydration solution, reduced infusion rate, monitoring for hypoglycemia and hypothermia) were applied instead of the standard protocols [8]. In this study, the characteristics of pediatric patients who were younger than 16 years and hospitalized at Heroes or Levy CTC were included for analysis.

### Data Collection

**Figure 1.**
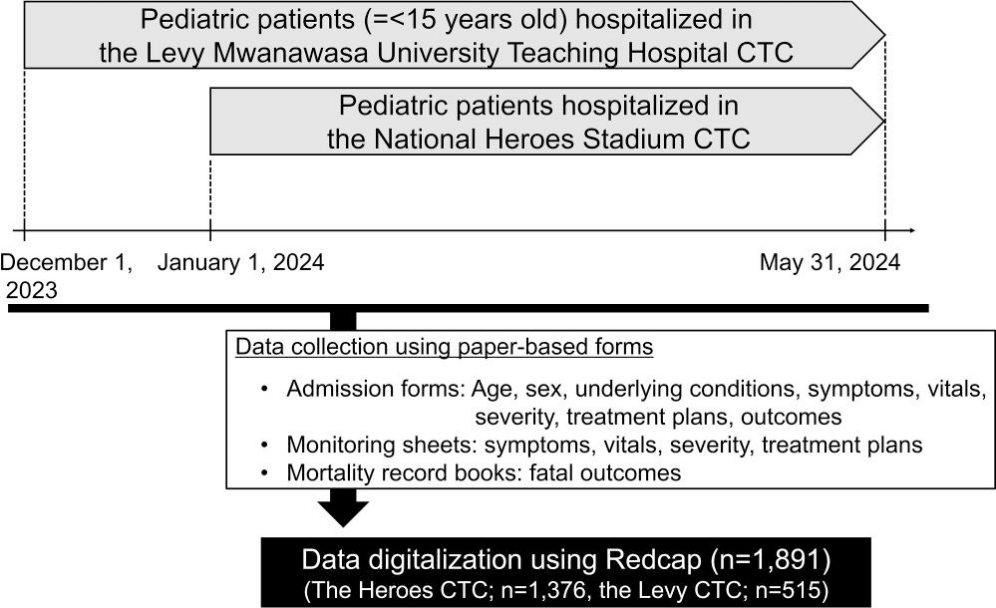
Study design. CTC, cholera treatment center.

Patient information recorded on admission forms and treatment progress forms adapted from GTFCC guidelines (Global Task Force on Cholera Control) and patient registers were retrospectively reviewed and digitalized (Figure 1) by trained data collectors and encoded into electronic data entry sheets developed with REDCap. Encoded data were exported as Microsoft Excel sheets and used as the master data set for analysis. Patients whose records were incomplete were censored in statistical analysis.

All data extracted from the records of patients were included in the study regardless of the missingness (Supplementary Table 1). The study consisted of records for patients aged 0 to 15 years who were treated at Heroes CTC and Levy CTC; to avoid duplicates, data were excluded from Heroes CTC for all patients seen at Heroes and subsequently referred to Levy CTC for comorbidities.

### Data Analysis

Descriptive analysis was conducted for patient characteristics that were recorded in the master data set. Length of hospitalization was calculated as the number of days between hospitalization and discharge. The day of hospitalization and discharge was counted as 1 day and included in the length of hospitalization. Hospitalization >2 days was regarded as a prolonged hospitalization based on the lengths of hospitalization (2–3 days) documented by the World Health Organization and the Médecins Sans Frontières and those reported in studies from other countries [9–12]. Continuous variables were summarized as median (IQR). Categorical values were summarized as the number (proportion) of patients with the characteristics. Temporal distribution of the pediatric patients who were included in the study was summarized in an epidemiologic curve based on the date of admission, with the moving average number of patients in the past 7 days.

During hospitalization, patients were regarded as having experienced clinical deterioration and an escalation of treatment plans when the plans were changed from A to B, A to C, and B to C and when patients had fatal outcomes after receiving treatment plan A, B, or C.

The CFR was calculated as the number of fatal cases divided by the total number of pediatric cases in the group. The CFR was calculated in total and for specific patient groups of interests (eg, ages, underlying conditions).

### Statistical Analysis

Statistical analysis was conducted in R version 3.5.0 (R Foundation for Statistical Computing). A χ^2^ test and a Fisher exact test, when appropriate, were used to compare patient characteristics in categorical variables between groups: first, between patients who were hospitalized for >2 days and ≤2 days; second, those who had fatal outcomes vs those who survived to hospital discharge. We performed the Student *t* test and the Wilcoxon rank sum test, when appropriate, to compare the characteristics of continuous variables between pediatric patients who were hospitalized for >2 and ≤2 days.

In the multivariate analysis, associations between outcomes were evaluated by Firth penalized logistic regression. Hospitalization >2 days and fatality served as the dependent variables, and patient characteristics such as age, sex, HIV positivity, presence of SAM signs, and treatment plan at admission served as the independent variables. Standard logistic regression was not adopted in this study because the models were not stable due to the small number of fatal cases. The degree of multicollinearity among independent variables in the model was evaluated by calculating the variance inflation factor for each variable. In addition, the difference in penalized log likelihood between each full model and null model was compared by a parametric bootstrapping method. Either full model improved the goodness of fit significantly from each null model (*P* < .001). The odds ratio (OR) and adjusted OR were calculated for binary and multivariate analysis, respectively, and presented with the 95% CI. *P* < .05 was considered statistically significant.

### Ethical Considerations

Institutional consent to use patient records was granted by the Ministry of Health.

Secondary use of the patient data, which were collected as part of the emergency medical response of the Ministry of Health and Zambia National Public Health Institute, was approved for analysis and publication by the National Health Research Authority (reference NHRA-1793/16/12/2024).

## RESULTS

### Patient Characteristics

**Figure 2.**
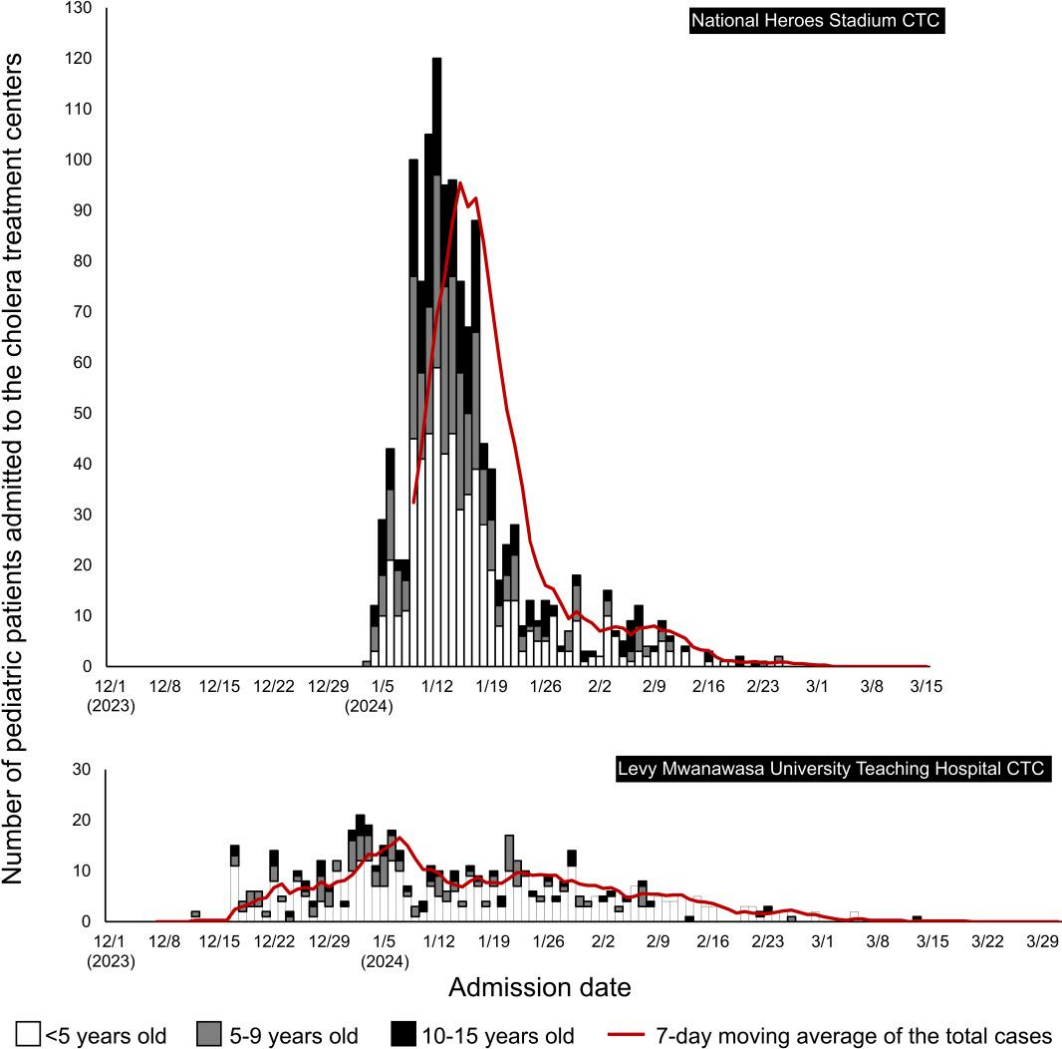
Temporal distribution of pediatric cases admitted to the referral cholera treatment centers in Lusaka, Zambia, between 1 December 2023 and 31 March 2024. CTC, cholera treatment center.

This study included 1891 pediatric patients aged 0 to 15 years. The temporal distribution of the patients shows that the number increased rapidly after the opening of Levy CTC on 1 December 2023 and Heroes CTC on 1 January 2024. In Heroes CTC, the number of patients reached its peak on 12 January 2024, although there were no such clear peaks in Levy CTC (Figure 2). In Heroes CTC, the number of new admissions decreased gradually toward late January, and 93.0% (1189/1279) of patients were hospitalized between 1 and 31 January.

The age category <5 years was most prevalent, representing 53.0% (1003/1891) of the total patient population (Table 1). The most prevalent underlying medical conditions were HIV infection (4.8%, 27/567), followed by SAM (3.1%, 59/1891). Among 546 patients, 34 (6.2%) had a history of oral cholera vaccine prior to admission. Median (IQR) days with diarrhea was 2.0 (1.0–2.0).

**Table 1.** Characteristics of 1891 Pediatric Patients Hospitalized at the Referral Cholera Treatment Centers in Lusaka, 1 December 2023–31 March 2024

		Hospitalization			Cases		
Characteristic	Pediatric Patients (n = 1891)	>2 d (n = 399)	≤2 d (n = 434)	*P* Value^a^	Fatal (n = 18)	Discharged (n = 1235)	*P* Value^a^
Age, y	4.0 (2.0–8.0)	3.0 (1.0–7.0)	4.0 (1.3–8.0)	<.001	1.0 (1.0–2.0)	4.0 (2.0–8.0)	<.001
Sex							
Female	839 (44.6)	177 (45.0)	180 (41.8)		8 (44.4)	542 (44.1)	
Male	1041 (53.4)	222 (55.6)	251 (58.2)	.493	10 (55.6)	686 (55.9)	>.99
Underlying medical conditions^b^							
HIV positive	27 (4.8)	18 (9.7)	4 (2.6)	.008	1 (20.0)	20 (4.4)	.208
Severe acute malnutrition^c^	59 (3.1)	41 (10.3)	11 (2.5)	<.001	5 (27.8)	47 (3.8)	<.001
Anemia	14 (2.7)	6 (2.5)	6 (3.1)	.773	1 (7.1)	9 (2.0)	.271
Epilepsy	3 (0.6)	3 (1.3)	0 (0)	.257	0 (0)	3 (0.7)	>.99
Tuberculosis	2 (0.4)	2 (0.8)	0 (0)	.505	0 (0)	2 (0.5)	>.99
Kidney diseases	1 (0.2)	1 (0.4)	0 (0)	>.99	0 (0)	1 (0.2)	>.99
Vaccination history							
Oral cholera vaccine prior to admission	34 (6.2)	4 (2.6)	3 (2.2)	>.99	0 (0)	24 (5.6)	>.99
History of present illness							
No. of days with diarrhea	2.0 (1.0–2.0)	2.0 (1.0–3.0)	2.0 (1.0–2.0)	.006	2.5 (2.0–3.0)	2.0 (1.0–2.0)	.071
Cholera treatment center							
National Heroes Stadium	1376 (72.8)	162 (40.6)	243 (56.0)		4 (22.2)	794 (64.3)	
Levy Mwanawasa University Teaching Hospital	515 (27.2)	237 (59.4)	191 (44.0)	<.001	14 (77.8)	441 (35.7)	.001

Data are presented as No. (%) of pediatric patients or median (IQR). Length of hospitalization was not available for 1058 cases, and clinical outcome (ie, fatality and discharge) was not available for 638 cases (Supplementary Table 1).

^a^χ^2^ test and Fisher exact test, when appropriate, were used to compare patient characteristics in categorical variables between 2 groups. We performed Student *t* test or Wilcoxon rank sum test, when appropriate, to compare patient characteristics in continuous variables between 2 groups.

^b^Anemia (n = 14), epilepsy (n = 3), tuberculosis (n = 2), and kidney diseases (n = 1).

^c^Defined as patients presenting mid upper arm circumference <11.5 cm, weight-for-height *z* score <−3, or bilateral pitting edema.

At admission, common clinical symptoms were sunken eyes (53.6%, 1013/1891), skin going back slowly after pinching (25.1%, 475/1891), and lethargy (15.1%, 285/1891; Table 2). Some dehydration (50.2%, 760/1515) was the most common severity, which was followed by no dehydration (35.6%, 539/1515) and severe dehydration (14.3%, 216/1515).

**Table 2.** Clinical Presentation, Diagnosis, Treatment, and Outcome of 1891 Pediatric Patients Hospitalized at the Referral Cholera Treatment Centers in Lusaka, 1 December 2023–31 March 2024

		Hospitalization			Cases		
Characteristic	Pediatric Patients (n = 1891)	>2 d (n = 399)	≤2 d (n = 434)	*P* Value^a^	Fatal (n = 18)	Discharged (n = 1235)	*P* Value^a^
Symptoms at admission							
Sunken eyes	1013 (53.6)	277 (69.4)	202 (43.5)	<.001	9 (50.0)	681 (55.1)	.812
Skin goes back slowly	475 (25.1)	159 (39.8)	94 (21.7)	<.001	7 (38.9)	328 (26.6)	.282
Lethargic	285 (15.1)	97 (24.3)	42 (9.7)	<.001	4 (22.2)	203 (16.4)	.520
Drinks eagerly	240 (12.7)	74 (18.5)	54 (12.4)	.019	2 (11.1)	166 (13.4)	>.99
Not drinking	151 (8.0)	47 (11.8)	25 (5.8)	.003	5 (27.8)	90 (7.3)	.009
Irritable	146 (7.7)	38 (9.5)	16 (3.7)	.001	3 (16.7)	97 (7.9)	.168
Unconscious	9 (0.5)	1 (0.3)	2 (0.5)	>.99	0 (0)	7 (0.6)	>.99
Vital signs at admission							
Temperature	36.5 (36.0–36.7)	36.3 (36.0–36.6)	36.5 (36.1–36.8)	<.001	37.0 (36.4–38.1)	36.5 (36.0–36.4)	<.001
Pulse rate	106.0 (96.0–120.0)	110.0 (98.0–124.0)	105.0 (94.0–122.0)	<.001	120.0 (106.0–132.5)	106.0 (95.0–120.0)	<.001
Respiratory rate	24.0 (21.0–30.0)	26.0 (22.0–30.0)	24.0 (20.0–30.0)	<.001	28.0 (24.0–30.0)	24.0 (20.0–28.0)	<.001
Systolic blood pressure	115.0 (102.0–123.0)	119.0 (107.0–125.0)	110.0 (100.0–117.0)	<.001	NA	116.0 (104.2–123.2)	NA
Diastolic blood pressure	76.0 (69.0–80.0)	77.0 (69.0–82.0)	75.5 (63.5–85.0)	<.001	NA	76.0 (69.0–80.0)	NA
SpO_2_	98.0 (95.0–98.0)	98.0 (97.0–99.0)	98.0 (96.0–98.0)	.003	98.0 (98.0–98.0)	98.0 (96.0–99.0)	.020
Disease severity at admission							
No dehydration	539 (35.6)	70 (19.6)	153 (43.5)		0 (0)	357 (35.0)	
Some dehydration	760 (50.2)	211 (59.1)	169 (48.0)		9 (64.3)	523 (51.3)	
Severe dehydration	216 (14.3)	76 (21.3)	30 (8.5)	<.001	5 (35.7)	139 (13.6)	.002
Initial treatment plan at admission							
A	554 (36.6)	74 (20.2)	156 (43.7)		0 (0)	350 (35.0)	
B	738 (48.7)	212 (57.9)	172 (48.2)		9 (69.2)	507 (50.6)	
C	222 (14.7)	80 (21.9)	29 (8.1)	<.001	4 (30.8)	144 (14.4)	.005
Clinical course during hospitalization							
Escalation of plan	253 (19.0)	68 (21.4)	30 (8.6)	<.001	3 (27.3)	148 (16.6)	.469
Outcomes							
Discharged	1235 (98.6)	379 (99.2)	389 (98.0)		NA	NA	NA
Fatal	18 (1.4)	3 (0.8)	8 (2.0)	.250	NA	NA	NA
Length of hospitalization	2.0 (2.0–4.0)	NA	NA	NA	2.0 (2.0–2.5)	2.0 (2.0–4.0)	.027
>2 d	399 (47.9)	NA	NA	NA	3 (27.3)	379 (49.3)	.224

Data are presented as No. (%) of pediatric patients or median (IQR). Length of hospitalization was not available for 1058 cases, and clinical outcome (ie, fatality and discharge) was not available for 638 cases (Supplementary Table 1).

Abbreviation: NA, not applicable.

^a^χ^2^ test and Fisher exact test, when appropriate, were used to compare patient characteristics in categorical variables between 2 groups. We performed Student *t* test or Wilcoxon rank sum test, when appropriate, to compare patient characteristics in continuous variables between 2 groups.

### Patient Treatment and Progress

Among 1514 pediatric patients, treatment plan A was applied at admission for 554 (36.6%), plan B for 738 (48.7%), and plan C for 222 (14.7%; Table 2). Among patients with severe dehydration, 87.5% (189/216) were administered treatment plan C at admission (Supplementary Table 2). A total of 18 fatal cases were reported, which represented a CFR of 1.4% (18/1253). The CFR was highest for the patient group with underlying conditions, such as SAM (8.5%), anemia (7.1%), and HIV (3.7%), followed by those hospitalized at Levy CTC (3.0%) and those diagnosed with severe dehydration (2.3%; Supplementary Table 3).

**Figure 3.**
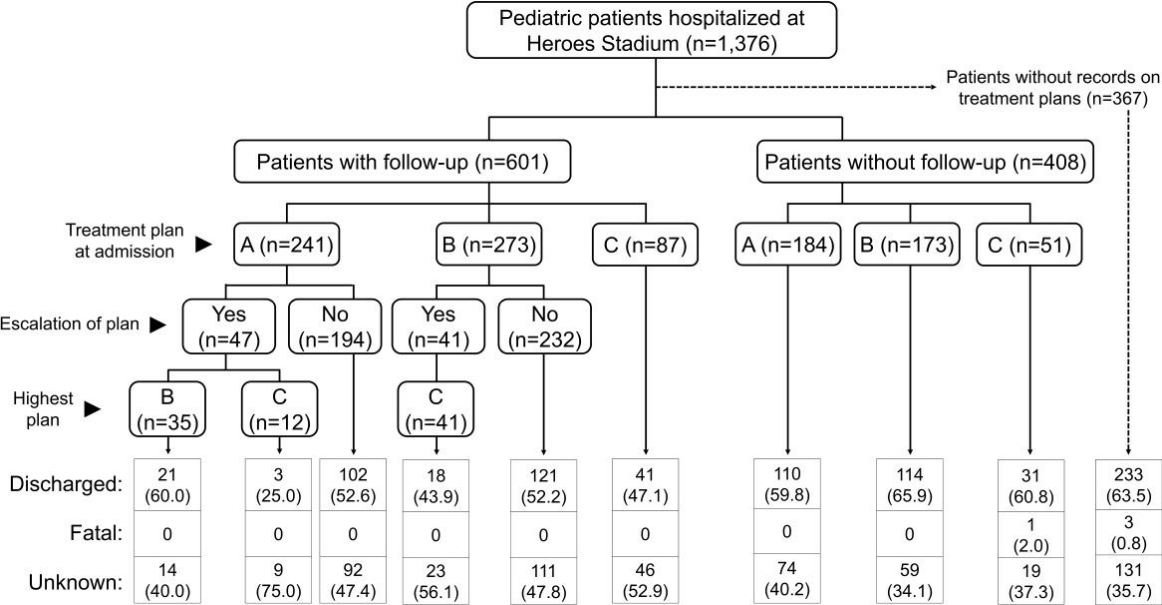
Clinical course of pediatric patients hospitalized at the National Heroes Stadium cholera treatment center in Lusaka, Zambia, between 3 January and 31 March 2024.

In Heroes CTC, records on patient follow-up were available for 601 cases. Among them, 47 cases with plan A and 41 cases with plan B, representing 14.6% (88/601), experienced an escalation of the treatment plans during hospitalization (Figure 3). However, all 306 cases with available records on the outcome survived to hospital discharge. Of 798 patients, 4 (0.5%) had fatal outcomes at Heroes CTC (Table 2).

**Figure 4.**
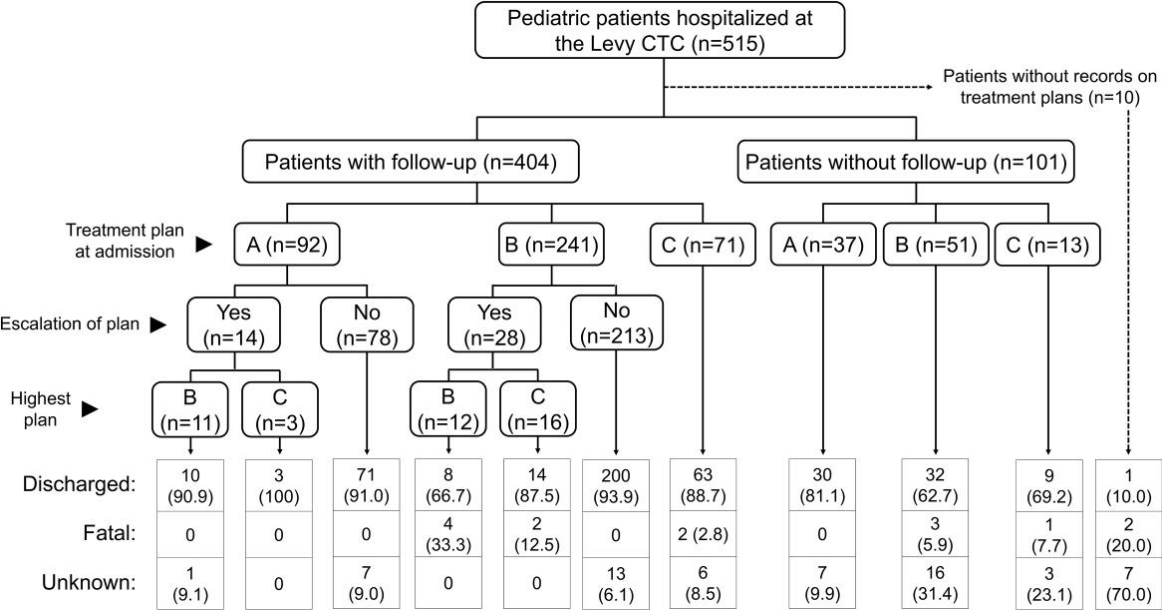
Clinical course of pediatric patients hospitalized at the Levy Mwanawasa University Teaching Hospital cholera treatment center in Lusaka, Zambia, between 1 December 2023 and 31 March 2024. CTC, cholera treatment center.

In Levy CTC, records on patient follow-up were available for 404 cases. Among them, 14 cases with plan A and 28 cases with plan B, representing 10.4% (42/404), experienced an escalation of the treatment plans during hospitalization (Figure 4). Although the majority of patients survived to hospital discharge, 14 had a fatal outcome at Levy CTC (Table 2). Among these 14 cases, 6 were initially assigned plan B and experienced an escalation of plans, and 2 were initially assigned plan C. The remaining 4 cases were initially assigned plan B or C, but records of their follow-up were not available.

### Patient Risk Factors and Outcome

In the unadjusted univariate analysis, the following were positively associated with hospitalization >2 days: comorbidities such as HIV infection (OR, 4.07; 95% CI, 1.48–14.33) and SAM (OR, 4.40; 95% CI, 2.31–9.12), CTC (OR, 1.86; 95% CI, 1.41–2.45), treatment plans B (OR, 2.60; 95% CI, 1.85–3.67) and C (OR, 1.76; 95% CI, 1.08–2.94) at admission, and an escalation of the treatment plan during hospitalization (OR, 2.77; 95% CI, 1.77–4.41; Table 3). For the fatal outcome, age (OR, 0.67; 95% CI, .49–.83) showed a negative correlation while SAM (OR, 9.72; 95% CI, 3.02–26.97) showed a positive correlation by univariate analysis.

**Table 3.** Factors Associated With Prolonged Hospitalization (>2 Days) Among 1891 Pediatric Patients Hospitalized at the Referral Cholera Treatment Centers in Lusaka, 1 December 2023–31 March 2024

	Hospitalization >2 d				Fatal Outcome			
	Crude		PLR^a^		Crude		PLR^a^	
Characteristic: Category	OR (95% CI)	*P* Value	OR (95% CI)	*P* Value	OR (95% CI)	*P* Value	OR (95% CI)	*P* Value
Age, y	0.98 (.95–1.01)	.167	0.99 (.91–1.07)	.737	0.67 (.49–.83)	.003	0.66 (.04–1.11)	.184
Sex								
Female	1 [Reference]		1 [Reference]		1 [Reference]		1 [Reference]	
Male	0.90 (.68–1.18)	.450	1.01 (.56–1.82)	.978	0.99 (.39–2.60)	.979	4.42 (.29–2.75e + 05)	.299
HIV								
No	1 [Reference]		1 [Reference]		1 [Reference]		1 [Reference]	
Yes	4.07 (1.48–14.33)	.013	6.89 (1.32–71.9)	.020	5.48 (.27–39.18)	.136	6.73 (.02–1.26e + 06)	.473
SAM^b^								
No	1 [Reference]		1 [Reference]		1 [Reference]		1 [Reference]	
Yes	4.40 (2.31–9.12)	<.001	10.8 (2.91–61.1)	<.001	9.72 (3.02–26.97)	<.001	0.61 (.00–18.6)	.835
Treatment plan at admission								
Plan A	1 [Reference]		NA		1 [Reference]		1 [Reference]	
Plan B	2.60 (1.85–3.67)	<.001	3.93 (1.80–9.27)	<.001	4.12 × 10^7^ (5.73 × 10^−43^–∞)	.991	2.99 (.23–2.61e + 05)	.452
Plan C	5.82 (3.54–9.78)	<.001	7.54 (2.71–22.9)	<.001	6.45 × 10^7^ (8.97 × 10^−43^–∞)	.991	1.60 (.01–1.37e + 05)	.825
Escalation of treatment plan during hospitalization								
No	1 [Reference]		1 [Reference]		1 [Reference]		1 [Reference]	
Yes	2.77 (1.77–4.41)	<.001	4.38 (1.10–25.3)	.035	1.46 (.37–4.52)	.547	1.81 (.01–40.5)	.731

The OR (95% CI) of pediatric patients with the characteristics is shown. The OR was calculated for binary analysis via a generalized linear model and PLR. *P* < .05 was considered statistically significant. Length of hospitalization was not available for 1058 cases, and clinical outcome (ie, fatality and discharge) was not available for 638 cases (Supplementary Table 1).

Abbreviations: OR, odds ratio; NA, not applicable; PLR, penalized likelihood regression; SAM, severe acute malnutrition.

^a^The PLR was used to evaluate the associations between outcomes, since the models using the standard logistic regression were not stable due to the small number of fatal cases.

^b^Defined as patients presenting mid upper arm circumference <11.5 cm, weight-for-height *z* score <−3, or bilateral pitting edema.

In the multivariate analysis with the penalized logistic regression, the following were independently correlated with hospitalization >2 days: comorbidities such as HIV infection (OR, 6.89; 95% CI, 1.32–71.9) and SAM (OR, 10.8; 95% CI, 2.91–61.1), treatment plans B (OR, 3.93; 95% CI, 1.80–9.27) and C (OR, 7.54; 95% CI, 2.71–22.9), and an escalation of the treatment plan during hospitalization (OR, 4.38; 95% CI, 1.10–25.3; Table 3). None of the variables showed any significant correlation with fatal outcome by the penalized logistic regression.

## DISCUSSION

The 2023–2024 cholera outbreak in Zambia is the largest in the country's history. Despite a high number of new cases and hospitalizations, the overall CFR at treatment centers remained <1%, even among pediatric patients. This is in contrast to the 2010 cholera outbreak in Haiti, which had a CFR >3% and nearly 10 000 deaths [13, 14]. In comparison with Zambia, Haiti had >820 000 cholera cases during the 2010 outbreak at a time when the country was still recovering from a major earthquake, which, with existing environmental and socioeconomic factors, likely contributed to the high morbidity and mortality [15]. From our research, no other cholera centers, aside from those in Haiti, have documented such a high volume of patients during a nonconflict period. The facility CFR for Levy CTC was 2.7% as compared with 0.3% for Heroes CTC, suggesting that having an underlying condition was a likely contributor, in line with findings from the GTFCC scoping review on cholera mortality by Pampaka et al [6], which identified comorbidities as risk factors for mortality. Children aged <5 years made up 94.4% of the mortality despite making up only 53% of the admissions and had an age-specific CFR of 1.7%, with increasing age negatively associated with mortality in univariate analysis. This is consistent with the greater burden of cholera positivity among children aged <5 years than any other age group in cholera-endemic countries in Asia and Africa [5].

Among patients with comorbidities, only SAM was associated with an increased risk of mortality in univariate analysis but not multivariate analysis. This might be due to the small number of fatal cases in the study population or to the confounding factors adjusted for. From literature we know that fluid management in cases of SAM can be challenging and that too much or too little fluid may contribute to mortality [16]. Indeed, for patients with SAM, diarrhea is a common presentation [17], and differentiating the effect of dehydration, the pathophysiologic effects of malnutrition [18], and fluid overload remains a challenge [18, 19]. The pathophysiologic changes seen in SAM require more cautious treatment of diarrhea and dehydration, as evidenced by the significantly less fluid and longer duration of rehydration as seen in World Health Organization–approved treatment plans [18]. Our data showed that a significant number of patients required more fluid resuscitation (plans B and C) among patients with comorbidities, SAM included, as compared with those without comorbidities, while still managing to have a modest CFR of 3.7% among those with SAM. The study therefore could not attribute the mortalities as being solely due to SAM but potentially due to greater severity of cholera in patients with SAM or to fluid overload. Studies into fluid requirements for cases of SAM have been few, and they have failed to demonstrate an increased risk among patients with SAM and greater fluid administration; in fact, in some cases, there is a suggestion of fluid depletion for patients undergoing current therapy [16]. Unfortunately, our study was unable to clearly differentiate deterioration of patients due to fluid overload.

In terms of duration of hospitalization, being assigned treatment plan B or C and having SAM and/or HIV independently showed a positive correlation with prolonged hospitalization. Most patients staying >2 days had signs of some or severe dehydration, representing at least 5% dehydration. These patients have the most profuse episodes of diarrhea, with fluid loss as much as 20 mL/kg/h in children [20], and diarrhea may persist even with improved hydration status. For SAM, the association was attributed to longer duration of symptoms of diarrhea, as well as longer fluid resuscitation regimens, in line with previous reports [21]. Having HIV was positively associated with prolonged hospitalization. Although studies have suggested an increased incidence of cholera among patients with HIV [22–24], this is one of the few reports that describes an association with longer duration of hospitalization. Further studies are needed to fully define this association in relation with the effect of vaccination and HIV treatment status (eg, viral load in DNA copies/mL, CD4 count) [22].

Approximately 15.3% of patients with fully documented treatment deteriorated from plan A or B to a higher treatment plan, and deterioration was associated with an increased probability of prolonged hospitalization in univariate analysis but not multivariate analysis. Deterioration was partly attributed to evolution of symptoms of cholera, with worsening or continued episodes of diarrhea for some. For others still, it was due to poor uptake of oral rehydration solution, a well-recognized challenge among pediatric patients, as well as poor monitoring for a few of the cases, particularly for plan A patients who tend to be monitored less frequently. Furthermore, some plan C patients were seen to require longer therapy with repeated plan C fluids before eventually improving, while others who improved at their next assessments later deteriorated, requiring further plan C (data not shown). This interesting observation prompted the need for more stringent monitoring and step-down rehydration therapy (plan B then A) for the recovering plan C patients. We strongly recommend further study and possible revision of guidelines on management of dehydration.

### Strengths and Limitations

The strength of this study lies in the large amount of the real-world data collected during the cholera outbreak from patient records and is among the few studies to date to focus on pediatric patients. We found no other study examining duration of hospitalization for patients with cholera of any age group or treatment progress for hospitalized patients with cholera.

Limitations include the retrospective study design, which is inherently susceptible to recall bias and potential missing information, as was the case with incomplete data entered into REDCap, which limited the number of patients in the statistical analysis. This was largely attributed to the overwhelmingly high number of patients for the limited number of health staff, especially in the initial stages of the outbreak. Of note is that our comparison of included and censored cases showed no significant difference, at least not in terms of demographic information.

In addition, as only patients with cholera were selected, there is an inherent selection bias, as cholera by its nature tends to be more common in communities of low socioeconomic status, and so our study did not investigate this association.

Last, this study has a lot of relatable aspects, with similar social, economic, and environmental risk factors present or coinciding with our outbreak, such as the El Nino experienced at the time. However, it may not be generalizable for all settings due to the unique nature of our context, which included a centralized approach to managing the outbreak and the availability of a huge pool of health personnel awaiting employment who volunteered to save lives.

## CONCLUSION

Despite a high influx of patients with cholera at 2 major treatment centers, teams managed to maintain a low CFR (<1%) among pediatric patients. The study found that comorbidities, especially SAM, and younger age were linked to higher mortality. Additionally, patients with comorbidities such as SAM and HIV, as well as those requiring more intensive treatment, faced longer hospital stays. The findings highlight the importance of improving fluid management, particularly for patients with underlying conditions such as SAM and HIV, to reduce morbidity and mortality.

## Supplementary Material

ofaf215_Supplementary_Data

## Supplementary Data

Supplementary materials are available at *Open Forum Infectious Diseases* online. Consisting of data provided by the authors to benefit the reader, the posted materials are not copyedited and are the sole responsibility of the authors, so questions or comments should be addressed to the corresponding author.

## Notes

***Acknowledgments.*** We thank all health care workers who supported the clinical management of cholera cases in Lusaka, Zambia, 2023–2024.

***Author contributions.*** N. Kalima and T. I. are the principal investigators. N. Kalima, T. I., C. C., K. Z., I. C., N. M., A. S., P. M. Z., K. Mwangilwa, M. K., A. Mugala, K. Mwewa, P. M., N. N., A. Mweemba, and L. M. designed the study. B. C. prepared the REDCap data collection forms. N. Kalima, T. I., and B. C. collected data. N. Kalima., T. I., S. M., Y. T., and T. K. analyzed data. S. M., Y. T., and T. K. provided critical support for statistical analysis. N. Kalima and T. I. prepared the manuscript. N. Kapata, R. C., and L. M. provided critical support for scientific presentation in the manuscript preparation.

***Data sharing.*** The data set used for this study will be shared by the study group with a reasonable request.

***Financial support.*** This work was supported by the Project for Strengthening Laboratory-Based Surveillance for Infectious Diseases, Japan International Cooperation Agency, to T. I.
