# Supplementary material for: Clinical Characteristics and Factors Associated With Severe Outcomes of 1891 Pediatric Patients Admitted to the Referral Cholera Treatment Centers in Lusaka, Zambia, December 2023–March 2024
Source: Open Forum Infect Dis. 2025 Apr 9;12(5):ofaf215. doi: 10.1093/ofid/ofaf215 (PMC12039484; doi:10.1093/ofid/ofaf215)
Supplement: ofaf215_Supplementary_Data [file ofaf215_supplementary_data.docx]

**Supplementary data**

Supplementary table 1. Number of patients hospitalized at the referral cholera treatment centers with available clinical data

| Characteristic | | Pediatric patients  (n=1,891)  [number (%)] | National Heroes Stadium  (n=1,376)  [number (%)] | Levy Mwanawasa University Teaching Hospital  (n=515)  [number (%)] |
| --- | --- | --- | --- | --- |
| Age | | 1,891 (100) | 1,376 (100) | 515 (100) |
| Sex | | 1,880 (99.4) | 1,365 (99.2) | 515 (100) |
| Underlying medical conditions | Any conditions | 963 (50.9) | 463 (33.6) | 500 (97.1) |
|  | Human immunodeficiency virus positives | 567 (30.0) | 309 (22.5) | 258 (50.1) |
|  | SAM* | 1,891 (100) | 1,376 (100) | 515 (100) |
| Oral cholera vaccine prior to admission | | 546 (28.9) | 345 (25.1) | 201 (39.0) |
| History of present illness | | 1,007 (53.3) | 527 (38.3) | 480 (93.2) |
| Symptoms at admission | | 1,891 (100) | 1,376 (100) | 515 (100) |
| Vitals at admission | Temperature | 794 (42.0) | 338 (24.6) | 456 (88.5) |
|  | Pulse rate | 683 (36.1) | 308 (22.4) | 375 (72.8) |
|  | Respiratory rate | 604 (31.9) | 247 (18.0) | 357 (69.3) |
|  | Systolic blood pressure | 49 (2.6) | 24 (1.7) | 25 (4.9) |
|  | Diastolic blood pressure | 45 (2.4) | 22 (1.6) | 23 (4.5) |
|  | SpO_2_ | 417 (22.1) | 217 (15.8) | 200 (38.8) |
| Disease severity at admission | | 1,515 (80.1) | 1,014 (73.7) | 501 (97.3) |
| Initial treatment plan at admission | | 1,514 (80.1) | 1,009 (73.3) | 505 (98.1) |
| Outcomes | | 1,253 (66.3) | 798 (58.0) | 455 (88.3) |
| Lengths of hospitalization | | 833 (44.1) | 405 (29.4) | 428 (83.1) |

* SAM; severe acute malnutrition, defined as patients presenting mid upper arm circumference < 11.5cm, or weight for height z-score < -3 or bilateral pitting oedema.

Supplementary Table 2. Assessed clinical severity and the initial treatment plan at admission among 1,891 pediatric patients hospitalized at the referral cholera treatment centers in Lusaka, between December 1, 2023 and March 31, 2024

| Treatment plan at admission | Clinical severity at admission | | | | |
| --- | --- | --- | --- | --- | --- |
|  | No dehydration | Some dehydration | Severe dehydration | Unknown | Total |
| A | 460 (85.3) | 54 (7.1) | 8 (3.7) | 32 (8.5) | 554 |
| B | 35 (6.5) | 665 (87.5) | 16 (7.4) | 22 (5.9) | 738 |
| C | 2 (0.4) | 19 (2.5) | 189 (87.5) | 12 (3.2) | 222 |
| Unknown | 42 (7.8) | 22 (2.9) | 3 (1.4) | 310 (82.4) | 377 |
| Total | 539 (100) | 760 (100) | 216 (100) | 376 (100) | 1,891 |

Number (%) of pediatric patients with the selected treatment plan at admission in each of the clinical severity at admission are shown.

Supplementary Table 3. Case fatality rate among 1,891 pediatric patients hospitalized at the referral cholera treatment centers in Lusaka, between December 1, 2023 and March 31, 2024

| Characteristic | Case fatality rate (%) | Number of fatal cases | Number of pediatric patients |
| --- | --- | --- | --- |
| Total | 1.4 | 18 | 1,253 |
| Facility |  |  |  |
| National Heroes Stadium | 0.5 | 4 | 798 |
| Levy Mwanawasa University Teaching Hospital | 3.0 | 14 | 455 |
| Age groups |  |  |  |
| <5 years old | 1.7 | 17 | 1,003 |
| 5-9 years old | 0.0 | 0 | 506 |
| 10-15 years old | 0.3 | 1 | 382 |
| Sex |  |  |  |
| Female | 1.0 | 8 | 839 |
| Male | 1.0 | 10 | 1,014 |
| Underlying conditions |  |  |  |
| Human immunodeficiency virus positives | 3.7 | 1 | 27 |
| SAM^+^ | 8.5 | 5 | 59 |
| Anemia | 7.1 | 1 | 14 |
| Epilepsy | 0.0 | 0 | 3 |
| Tuberculosis | 0.0 | 0 | 2 |
| Kidney diseases | 0.0 | 0 | 1 |
| Oral cholera vaccine prior to admission |  |  |  |
| Yes | 0.0 | 0 | 34 |
| No | 1.4 | 7 | 512 |
| Initial diagnosis at admission |  |  |  |
| No dehydration | 0.0 | 0 | 539 |
| Some dehydration | 1.2 | 9 | 760 |
| Severe dehydration | 2.3 | 5 | 216 |
| Initial treatment plan at admission |  |  |  |
| A | 0.0 | 0 | 554 |
| B | 1.2 | 9 | 738 |
| C | 1.8 | 4 | 222 |

+ SAM; severe acute malnutrition, defined as patients presenting mid upper arm circumference < 11.5cm, or weight for height z-score < -3 or bilateral pitting oedema.
